# Research on the relationship between fibrinogen level and subtypes of the TOAST criteria in the acute ischemic stroke

**DOI:** 10.1186/1471-2377-13-207

**Published:** 2013-12-19

**Authors:** Qi Lang, Muke Zhou, Hong Feng, Jian Guo, Ning Chen, Li He

**Affiliations:** 1 Department of Neurology, West China Hospital, Sichuan University, Chengdu 610041, China

**Keywords:** Fibrinogen, Ischemic stroke, Subtypes of stroke, TOAST Criteria, Correlation

## Abstract

**Background:**

Cerebral infarction caused by different reasons seems differ in fibrinogen levels, so the current work intends to explore the relationship between the fibrinogen level and subtypes of the TOAST criteria in the acute stage of ischemic stroke.

**Methods:**

A total of 577 case research objects were treated acute ischemic stroke patients in our hospital from December 2008 to December 2010, and blood samples within 72 hours of the onset were processed with the fibrinogen (PT-der) measurement. Classification of selected patients according to the TOAST Criteria was conducted to study the distribution of fibrinogen levels in the stroke subtypes.

**Results:**

The distribution of fibrinogen levels in the subtypes was observed to be statistically insignificant.

**Conclusions:**

In the acute stage of ischemic stroke, fibrinogen level was not related to the subtypes of the TOAST criteria.

## Background

Currently, the classification of strokes according to the TOAST Criteria [1] is the most widely used etiologic approach for ischemic stroke classification. The approach classifies ischemic stroke into five subtypes: large-artery atherosclerosis (LAA), cardioembolism (CE), small-artery occlusion lacunar (SAO), stroke of other demonstrated etiology (SOE), and stroke of other undemonstrated etiology (SUE). The classification method based on the TOAST Criteria mainly depends on the assistant examinations, such as ultrasound and images. In some special cases, the classification of the patients using the TOAST Criteria is extremely difficult. For example, when the patient cannot complete the imaging examinations or the etiology cannot be determined because several etiologic factors are found through imaging and ultrasound. If changes in some indicators in the blood can suggest the etiology of ischemic strokes, it can be very helpful for the etiologic classification of ischemic stroke. Studies have found that cerebral infarction caused by large vascular disease generates higher fibrinogen (Fib) levels than that induced by small vascular disease [2]. The relationship between the fibrinogen level and subtypes of the TOAST criteria in the acute stage of ischemic stroke is thus worth exploring.

## Methods

### Research object

Our research was a retrospective study which consecutively included 577 patients suffering from acute cerebral infarction who were treated at the Department of Neurology, West China Hospital of Sichuan University, from December 2008 to December 2010 within 72 hours of the onset. The diagnoses were consistent with the revised diagnostic criteria by the Fourth Chinese National Cerebrovascular Disease Conference [3], which was modified from the standard WHO definition of ischemic stroke. Patient information was collected, including the medical history, blood routine examination, urination and defecation routine examination, blood lipids, coagulability, liver and kidney function, electrolytes, glucose, Immunologic test, ECG, vascular ultrasound, CTA or MRA, DSA if available and other tests. The study protocol was approved by the Local Ethics Committee of West China Hospital, Sichuan University.

### Research methods

#### Specimen collection

All specimens were selected from blood samples within 72 hours from the onset of the disease, and all test specimens were measured within 1 day of the blood collection. The test device was an ACL-9000 and the PT-der approach was adopted.

### Classification of stroke

Based on clinical manifestation and complete auxiliary examination of patients, all the cases of cerebral infarction were divided into five subtypes according to the TOAST criteria: large-artery atherosclerosis (LAA), cardioembolism (CE), small-artery occlusion lacunar (SAO), stroke of other determined etiology (SOE), and stroke of undetermined etiology (SUE). Two neurologist independently subtyped the ischemic strokes, and differences in opinion were resolved by discussion.

Inclusion criteria: Diagnosis of ischemic stroke according to the Fourth Chinese National Cerebrovascular Disease Conference 2、 within 72 hours after the onset 3、 a relatively accurate TOAST classification could be made according to given clinical materials.

Exclusion criteria: Since Fibrinogen is also a marker of systemic inflammation, our exclusion criteria are 1. Patients with infection within 3 months or untreated chronic infections; 2. Patients with connective tissue diseases, 3. Subjects treated with any thrombolysis, anticoagulation, defibrase or antiplatelet agents, 4. Pregnant females, 5. Subjects with serious systematic disorders.

### Statistical methods

Due to the inconformity of normal distribution, data is presented as the median. SPSS 13.0 statistical software was used for statistical analysis. The relationship among samples was analyzed with a Kruskal-Wallis analysis of variance test. *P* < 0.05 is viewed as significant difference.

## Results

The demographic data: 577 patients were included, including 328 males and 249 females, with an average age of 67 years (the lower limit of quartile was 57, and the upper limit of the quartile was 74. Quartile range was 17).

2.2 Subtype distribution of the TOAST criteria: there were 185 cases (32.06%) of LAA, 126 cases (21.84%) of CE, 214 cases (37.09%) of SAO, 26 cases of SOE (4.51%), and 26 cases of SUE (4.51%), and it is consistent with the report ofDirk Deleu [4]. The distribution of risk factors such as smoking, alcohol consumption, hypertension, diabetes, stroke history, and lipid levels between the subtypes is shown in Table 1.

**Table 1:** Distribution of demography in stroke patients classified TOAST subtype

**Subtypes of TOAST criteria**	**CE**	**LAA**	**SAA**	**SOE**	**SUE**
Stroke distribution (N, SE)	126 (21.8)	185 (32.1)	214 (37.1)	26 (4.5)	26 (4.5)
Age (Y,SE)	70 (12.1)	67 (11.6)	66 (11.4)	47 (8.1)	67 (11.6)
Gender (N, SE)					
Female	43 (7.5)	114 (19.8)	137 (23.7)	17 (2.9)	17 (2.9)
Male	83 (14.4)	71 (12.3)	77 (13.3)	9 (1.6)	9 (1.6)
Smoking (N, SE)	18 (3.1)	63 (10.9)	66 (11.4)	8 (1.4)	8 (1.4)
Alcohol consumption (N, SE)	7 (1.2)	42 (7.3)	41 (7.1)	7 (1.2)	7 (1.2)
Hypertension (N, SE)	39 (6.8)	87 (15.1)	118 (20.5)	8 (1.4)	10 (1.7)
Diabetes (N, SE)	19 (3.3)	45 (7.8)	57 (9.9)	2 (0.3)	7 (1.2)
Stroke history (N, SE)	13 (2.3)	14 (2.4)	24 (4.2)	0 (0)	2 (0.3)
Lipid (mmol/L, SE)					
TG	1.11 (0.8)	1.69 (0.9)	1.83 (1.1)	1.89 (0.8)	1.56 (1.2)
CHOL	3.95 (1.6)	4.65 (1.3)	4.69 (1.5)	4.4 (1.2)	4.41 (1.6)
HDL	1.3 (0.5)	1.32 (0.5)	1.3 (0.5)	1.37 (0.4)	1.22 (0.5)
LDL	2.29 (1.2)	2.87 (1.2)	2.82 (1.1)	2.48 (1.0)	2.76 (1.5)
Fibrinogen (g/L, SE)	2.94 (0.9)	3.02 (1.1)	2.9 (1.1)	2.84 (0.6)	3.12 (1.1)

Fibogen value distribution of the TOAST criteria: CE: 2.94 g/L; LAA: 3.02 g/L; SAO: 2.90 g/L; SOE: 2.84 g/L; SUE: 3.12 g/L (the lower limit of quartile was 2.42, and the upper limit of quartile was 3.43. Quartile range was 1.01).

The Kruskal-Wallis analysis of variance test found *p* = 0.576 so that the value of fibrinogen among various subtypes showed no significant difference.

According to the median gap, fibrinogen levels were divided into the following four groups (0–2.42, 2.42 - 2.88, 2.88 - 3.43, and 3.43 - 7.13). Fibrinogen distribution among subtypes is shown in the following table (Table 2).

**Table 2:** Fibrinogen distribution among subtypes

**Fibrinogen (g/L)**				
	**0–2.42**	**2.42–2.88**	**2.88–3.43**	**3.43–7.13**
CE (n/%)	27 (21.43%)	34 (26.98%)	40 (31.75%)	25 (19.84%)
LAA (n/%)	46 (24.86%)	40 (21.62%)	43 (23.24%)	56 (30.27)
SAA (n/%)	59 (27.57%)	53 (24.77%)	48 (22.43%)	54 (25.23%)
SOE (n/%)	7 (26.92%)	8 (30.77%)	8 (30.77%)	3 (11.54%)
SUE (n/%)	5 (19.23%)	7 (26.92%)	5 (19.23%)	9 (34.62%)

Based on the Kruskal-Wallis analysis of variance test analysis, *p* = 0.496 so that the level of fibrinogen among the various subtypes showed no significant difference.

### Multivariate analysis

SUE and SOE were merged into one type because the case number of both types is smaller. Factors such as blood pressure, diabetes, stroke history, smoking history, alcohol consumption history, lipid, gender, fibrinogen levels, were included in the logistic regression analysis. Gender: CE was considered as the control, and other subtypes were compared with it. The difference in gender distribution was statistically significant, and the proportion of males in each type was lower than that in CE. Blood pressure: CE was considered as the benchmark. The hypertension among LAA and SAO patients was more severe than that in CE, whereas the hypertension in SUE and SOE showed no significant difference compared with that of CE. Lipids: CE was considered as the benchmark. The lipid levels of other subtypes were higher than that in CE. The levels of diabetes, stroke history, smoking, alcohol consumption, and fibrinogen in all subtypes showed no statistical significance.

## Discussion

Currently, many studies have shown that the soaring fibrinogen levels have a significant relationship with ischemic stroke [5,6], but few studies have been conducted on the relationship between fibrinogen and stroke subtypes. Yang D et al. have found that in the atherosclerosis type, the fibrinogen level is higher than that in the lacunar type of stroke [7]; however, only 85 patients were selected for classification using the TOAST criteria. Consequently, analysis of a larger sample size was conducted. Up to 577 patients who suffered from ischemic strokes were classified according to the TOAST criteria. Comparative analysis of the fibrinogen levels in the subtypes, and the distribution of fibrinogen level in various subtypes were carried out, which revealed that the fibrinogen levels in different TOAST subtypes were not statistically different. Fibrinogen is not related to the various subtypes in the TOAST criteria, which is consistent with the opinion proposed by Peter M. Rothwell and K. JOOD. In the report, fibrinogen showed no significant association with infarction of atherosclerotic thrombosis and non-lacunar stroke [2,8].

We consider that fibrinogen is not only inflammatory, but also relevant with thrombosis, the formation of atherosclerotic plaque as well as platelet aggregation involved during strokes at different levels. Thus, in any types of stroke, such as cardiac stroke, artery atherosclerosis stroke or other types, the fibrinogen level may be increased. The study of Alvarez-Perez [9] holds the same view with this paper. In their study of 200 cases of samples, the highest median of fibrinogen was found in patients with cardioembolic stroke followed by patients with atherothrombotic stroke, but differences among subtypes were nou significant. It means fibrinogen level is shown to increase in all the subtypes of stroke. However, in all subtypes, fibrinogen levels showed no significant difference. Furthermore, fibrinogen is related to inflammation, thrombosis, atherosclerotic plaque formation, and platelet aggregation: in CE, the increase in fibrinogen levels is mainly related to thrombosis; in SAO and SUE, the increase in fibrinogen is not specific. In LAA, the increase in fibrinogen is mainly related to the inflammatory response.

A study also found that CRP is related to LAA in some studies [10]. In the inflammation process, fibrinogen and CRP levels are closely linked [11]; also supporting the idea is the significant relationship of fibrinogen to the inflammatory response in the atherosclerotic type. Additionally, Lominadze [12] mentioned in his article that as one of the inflammatory factors, an increase in fibrinogen can lead to increased red blood cells and platelets, resulting in thrombosis. Fibrinogen simultaneously plays a variety of roles during strokes. The effects cannot be distinguished by fibrinogen. Therefore, the fibrinogen level in all subtypes of stroke show no significant differences. However, due to the limited conditions, the PT-der approach was used when detecting fibrinogen. When the concentration of fibrinogen was less than 1 g/L and higher than 4 g/L, the differences [13] were found comparable to those with the approach recommended by Von Clause [14], which may lead to errors. A larger multi-ceter experiments with more precise test and larger sample sizes are needed.

In multivariate regression, the stroke history, diabetes, smoking, alcohol consumption, and fibrinogen in all subtypes were not statistically significant. Yet, gender, blood pressure, and lipid levels among different subtypes of stroke show differences in distribution. In terms of the gender ratio, the male proportion of other types was lower than that of CE, which is consistent with the conclusions of Friberg [15]. This is relevant, given that more men smoke and drink alcohol. Hypertension among LAA and SAO patients was higher than that of CE, which is consistent with the TOAST classification criteria. Ohira et al. [16] considered that in various subtypes, the hypertension level does not change because of the classification criteria. Another way those with a positive family history of stroke have 30% increased risk of stroke according to some studies [17], for patients with TIA and stroke histories, the risk of another stroke is linearly linked with fibrinogen levels. Yet, other studies have a contrasting conclusion [8]. However, the current study indicates that the history of stroke is not related to stroke subtypes. The type of a second stroke cannot be determined based on the previous one. Smoking and alcohol consumption, with respect to gender differences, have proven unrelated to stroke subtypes.

## Conclusions

According to the results of the present study, fibrinogen level was not related to the subtypes of the TOAST criteria in the acute stage of ischemic stroke.

## Competing interests

The authors declare that they have no competing interests.

## Authors’ contributions

LQ and ZM collected participants and relevant data, and drafted the manuscript. GJ and CN assessed participants and made etiological classifications. FH conducted statistical analyses. HL supervised the study conduction and revised the manuscript. All authors read and approved the final manuscript.

## Pre-publication history

The pre-publication history for this paper can be accessed here:

http://www.biomedcentral.com/1471-2377/13/207/prepub
